# Analysis of Nonlinear Thermoelastic Dissipation in Euler-Bernoulli Beam Resonators

**DOI:** 10.1371/journal.pone.0164669

**Published:** 2016-10-13

**Authors:** Zahra Nourmohammadi, Surabhi Joshi, Srikar Vengallatore

**Affiliations:** Department of Mechanical Engineering, McGill University, Montreal, Quebec, Canada; University of Zaragoza, SPAIN

## Abstract

The linear theory of thermoelastic damping (TED) has been extensively developed over the past eight decades, but relatively little is known about the different types of nonlinearities that are associated with this fundamental mechanism of material damping. Here, we initiate the study of a dissipative nonlinearity (also called thermomechanical nonlinearity) whose origins reside at the heart of the thermomechanical coupling that gives rise to TED. The finite difference method is used to solve the nonlinear governing equation and estimate nonlinear TED in Euler-Bernoulli beams. The maximum difference between the nonlinear and linear estimates ranges from 0.06% for quartz and 0.3% for silicon to 7% for aluminum and 28% for zinc.

## Introduction

Thermoelastic damping (TED) refers to energy dissipation due to irreversible heat conduction across thermoelastic temperature gradients in vibrating structures [1, 2]. The foundations of the linear theory of TED were established in the 1930s, and developed extensively over the past thirty years (see, for instance [3–5] for reviews). The linear analysis is now widely used to gain insight into a fundamental mechanism of material damping, calibrate measurements of internal friction in thin films, and guide the design of miniaturized resonators used in microelectromechanical systems (MEMS).

In stark contrast, nonlinear thermoelastic dissipation has received relatively little attention. The literature is sparse and focuses mainly on the following three types of nonlinearities: (i) geometric nonlinearities due to large deformations, (ii) material nonlinearity due to the temperature dependence of mechanical properties, and (iii) transduction nonlinearities in oscillators caused by electrostatic actuation [6–15]. In this paper, we address a fourth type of nonlinearity whose origins reside at the heart of the thermoelastic coupling that gives rise to dissipation. For this reason, it may be termed a dissipative nonlinearity (or thermoelastic nonlinearity).

To understand the source of the dissipative nonlinearity, let us consider the time-harmonic oscillations of a thermoelastic structure that is initially at equilibrium at temperature *T*_0_. Due to thermoelastic coupling, the bending vibrations create a time-dependent temperature field *T* within the structure. In turn, the temperature gradients lead to irreversible heat conduction, entropy generation, and energy dissipation [1].

For diffusive heat conduction according to Fourier’s law, the temperature is governed by a nonlinear partial differential equation (PDE) given by [16]

$$C\frac{\partial \theta }{\partial t}=k\nabla^{2}\theta -\frac{E\;\alpha \;T}{\left(1-2\upsilon \right)}\frac{\partial \varepsilon_{nn}}{\partial t},$$ (1)

where *θ* = *T*−*T*_0_ is the excess temperature, *C* is the specific heat per unit volume, *t* is time, *k* is the thermal conductivity, *E* is Young’s modulus, *α* is the coefficient of thermal expansion, *υ* is Poisson’s ratio, and *ε* is the total strain (that is, the sum of elastic strain and thermal strain). The summation convention is implied for the index *n*; hence, *ε*_*nn*_ denotes the trace of the strain tensor.

The second term on the right-hand side of Eq (1) gives rise to the dissipative nonlinearity. Thus far, this effect has been largely ignored in the literature, and the governing equation is usually linearized by replacing *T* with *T*_0_. The error caused by linearization is expected to be small, but this important assumption has yet to be rigorously examined and quantified. In the remaining sections of the paper, we present a detailed numerical analysis of nonlinear TED and quantify the error incurred by ignoring the dissipative nonlinearity.

## Methods

Consider an isotropic, homogeneous, Euler-Bernoulli beam of length *L*, thickness *h*, and width *b*. A Cartesian coordinate system (*x*,*y*,*z*) is attached to the structure so that the beam occupies the domain defined by 0 ≤ *x* ≤ *L*, 0 ≤ *y* ≤ *b*, and 0 ≤ *z* ≤ *h*. The axial stress *σ*_*xx*_ = *σ*_0_ exp(i*ωt*) is the only nonzero component of the stress tensor; here, *ω* is the oscillation frequency (in units of radians per second) and $\text{i}=\sqrt{-1}$. The oscillatory curvature of the beam under pure bending is *κ*_*xx*_ = *z*_0_ exp(i*ωt*), where *z*_0_ is the amplitude of oscillation. The normal strains are given by [16]

$$\begin{array}{l} \varepsilon_{xx}=\frac{\sigma_{xx}}{E}+\alpha \theta =-\left(z-\frac{h}{2}\right)\kappa_{xx}\\ \varepsilon_{yy}=\varepsilon_{zz}=\frac{-\upsilon }{E}\sigma_{xx}+\alpha \theta \end{array}$$ (2)

and *z* = *h*/2 is the position of the neutral axis.

Using the expressions for the stresses and strains, and for one-dimensional heat conduction across the thickness of the beam, Eq (1) can be expressed as

$$C\left(1+2\Psi \frac{1+\upsilon }{1-2\upsilon }\right)\frac{\partial \theta }{\partial t}=k\frac{\partial^{2}\theta }{\partial z^{2}}+\text{i}E\alpha \;\left(\theta +T_{0}\right)\;\omega_{n}\;z_{0}\left(z-h/2\right)\exp\left(\text{i}\omega_{n}\;t\right)-2\left(\frac{1+\upsilon }{1-2\upsilon }\right)E\alpha^{2}\theta \frac{\partial \theta }{\partial t}$$ (3)

where Ψ = *Eα*^2^*T*_0_/*C* is the dimensionless Zener modulus. The beam is initially at equilibrium at temperature *T*_0_ and the boundaries along the *z*-direction are adiabatic; hence, the initial and boundary conditions are given by

$$\theta =0\;\text{at}\;t=0;\;\frac{\partial \theta }{\partial z}=0\;\text{at}\;z=0;\;\frac{\partial \theta }{\partial z}=0\;\text{at}\;z=h$$ (4)

We employ the method of finite difference to solve the nonlinear partial differential equation, Eq (3), subject to the initial and boundary conditions expressed in Eq (4). The first step is to cast the PDE in dimensionless form by defining the following variables

$$\bar{\theta }=\frac{\theta }{\theta_{c}},\;\theta_{c}=\frac{E\alpha \;h\;z_{0}T_{0}}{C};\;\bar{t}=\frac{t}{t_{c}},\;t_{c}=\frac{Ch^{2}}{k},\;\Omega =\omega t_{c};\;\bar{z}=\frac{z}{h}$$ (5)

Here, $\bar{\theta }$ is the normalized temperature, $\bar{t}$ is the normalized time, $\bar{z}$ is the normalized coordinate, and Ω is the normalized frequency. Hence, Eq (3) can be expressed in dimensionless form as

$$\left(1+\beta \right)\frac{\partial \bar{\theta }}{\partial \bar{t}}=\frac{\partial^{2}\bar{\theta }}{\partial {\bar{z}}^{2}}-\beta \;\lambda \;\bar{\theta }\frac{\partial \bar{\theta }}{\partial \bar{t}}+\text{i}\;\Omega \left(\bar{z}-0.5\right)\exp\left(\text{i}\;\Omega \bar{t}\right)\left(1+\lambda \bar{\theta }\right),$$ (6)

where

$$\beta =2\Psi \left(\frac{1+\upsilon }{1-2\upsilon }\right)\;\text{and}\;\lambda =\frac{\theta_{c}}{T_{0}}.$$ (7)

Eq (6) was solved using the implicit Crank-Nicholson finite-difference scheme with discretization grids that are uniform in space ($\bar{z}$) and time ($\bar{t}$) [17]. All the derivatives are replaced by the corresponding finite difference approximation to get

$$\begin{array}{l} \left[1+2N+\beta -0.5\lambda M\right]{\bar{\theta }}_{j}^{n+1}-N\left({\bar{\theta }}_{j-1}^{n+1}+{\bar{\theta }}_{j+1}^{n+1}\right)\\ =N\left({\bar{\theta }}_{j-1}^{n}+{\bar{\theta }}_{j+1}^{n}\right)+\left[1-2N+\beta +0.5\lambda M\right]{\bar{\theta }}_{j}^{n}+M-0.5\;\beta \;\lambda \left({\bar{\theta }}_{j}^{n+1}+{\bar{\theta }}_{j}^{n}\right)\left({\bar{\theta }}_{j}^{n+1}-{\bar{\theta }}_{j}^{n}\right) \end{array},$$ (8)

where $N=0.5\Delta \bar{t}/\Delta {\bar{z}}^{2}$ and $M=\text{i}\;\Omega \Delta \bar{t}\left(\bar{z}-0.5\right)\exp\left(\text{i}\Omega \bar{t}\right)$. The superscripts *n* + 1 and *n* denote the temperature at the current and previous iteration, respectively, and the subscripts refer to the nodes of the finite-difference mesh. This equation was expressed in matrix form and solved iteratively using the tri-diagonal matrix algorithm to obtain the temperature field. Finally, by definition, the nonlinear thermoelastic damping is given by [3, 18]

QTED−1=12πΔWW=12π24Ez02h3b∮∫VRe(σxx)Re(ε˙th)dVdt. (9)

Here, *V* is the volume of the beam, ∮ denotes the integral over one cycle of oscillation, *ε*^*th*^ = *αθ* is the thermal strain, and the peak elastic energy in the beam is $W=Ez_{0}^{2}h^{3}b/24$. Taken together, the aforementioned equations constitute a framework for computing nonlinear thermoelastic dissipation in Euler-Bernoulli beam resonators. The framework was implemented using Matlab to analyze nonlinear TED and the results are presented in the next section.

## Results

Nonlinear TED was analyzed in Euler-Bernoulli beams made of five different materials: quartz, silicon, diamond-like carbon (DLC), aluminum, and zinc. The materials were chosen to span the range of thermomechanical properties pertinent to TED. Table 1 lists the effective isotropic material properties at room temperature.

**Table 1:** Thermomechanical material properties at 300 K [19].

Material	*E* (GPa)	*k* (W/m/K)	*α* (K^-1^)	*C* (J/m^3^/K)	Ψ = *Eα*^2^*T*_0_ / *C*
Quartz	70	1.2	5×10^−7^	1.5×10^6^	3.5×10^−6^
Silicon	160	150	2.6×10^−6^	1.6×10^6^	2.0×10^−4^
Diamond-like carbon (DLC)	800	800	1.5×10^−6^	2.4×10^6^	2.2×10^−4^
Aluminum	70	220	2.4×10^−5^	2.4×10^6^	5.0×10^−3^
Zinc	100	110	4.0×10^−5^	2.7×10^6^	1.8×10^−2^

In all cases, the nonlinear estimates were compared with the prediction of the linear theory of TED in Euler-Bernoulli beams given by [20]

$$Q_{\text{TED},\;\text{Linear}}^{-1}=\Psi \frac{6}{\xi^{2}}\left\{1-\frac{\sinh\xi +\sin\xi }{\xi \left(\cosh\xi +\cos\xi \right)}\right\};\;\xi =h\sqrt{\frac{\omega C}{2k}}.$$ (10)

**Fig 1:**
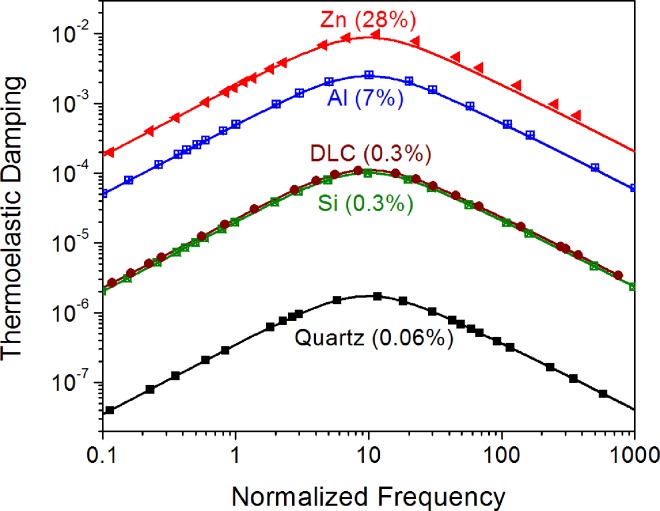
Frequency dependence of thermoelastic damping for Euler-Bernoulli beams of quartz, silicon, diamond-like carbon (DLC), aluminum, and zinc. The symbols are the results of the numerical analysis of nonlinear TED, and the lines are the predictions of the linear model given by Eq (10). The numbers in parentheses denote the maximum difference between the nonlinear and linear estimates for TED.

The main results are presented in Fig 1 on a graph of TED as a function of the normalized frequency (Ω = *ωCh*^2^ / *k*). The symbols denote the results from the numerical analysis of nonlinear TED, and the lines are the predictions of the linear model given by Eq (10).

The dissipative nonlinearity does not introduce any new spectral features. In common with the linear model, nonlinear TED also predicts a single dissipation peak with a maximum value of 0.5Ψ at a critical normalized frequency of Ω^*^ = *π*^2^. For both linear and nonlinear analysis, the magnitude of the dissipative nonlinearity is independent of the oscillation amplitude. Both models are in excellent quantitative agreement below the critical frequency (Ω < Ω^*^). However, for Ω > Ω^*^, the nonlinear analysis predicts a higher value for TED. The maximum difference between the nonlinear and linear estimates is 0.06% for quartz, 0.3% for both silicon and diamond-like carbon, 7% for aluminum, and 28% for zinc. Taken together, the results indicate that the error caused by ignoring the dissipative nonlinearity scales with the Zener modulus of the material.

## Discussion

Thermoelastic damping arises as a consequence of the nonlinear coupling between thermal and elastic fields in vibrating solids. In this paper, we presented the first detailed numerical analysis of the effects of the dissipative nonlinearity on thermoelastic damping in Euler-Bernoulli beams. All other types of nonlinearities (geometric, material, and transduction) were eliminated from the analysis, and the effects of the dissipative nonlinearity were quantified by comparison with a linear model for thermoelastic damping.

Results were presented for five representative materials that span the full range of the Zener modulus encountered in metals, alloys, glasses, and ceramics. The dissipative nonlinearity does not introduce any new spectral features, and the dissipation spectra display a single peak. The error caused by ignoring the dissipative nonlinearity is negligible at below the peak frequency for all materials. Thus, the linear approximation can be used with confidence for design and analysis in this regime. However, beyond the peak frequency, the error is proportional to the Zener modulus of the material. The maximum error can be as high as 7% for aluminum and 28% for zinc.

To our knowledge, the present study is the first to quantify the effects of the dissipative nonlinearity on thermoelastic damping. The analysis focused on isotropic and homogeneous Euler-Bernoulli beams, and ignored the effects of material and geometric nonlinearities. Our results suggest several fruitful and intriguing topics for future investigations including the study of nonlinear thermoelastic dissipation in other structures (plates, hollow tubes, rings, shells, and layered composites) and anisotropic materials, and exploring the interactions between material, geometric, and dissipative nonlinearities. A useful starting point for such studies is to replace Eq (1) with a fully nonlinear equation for thermoelastic heat conduction in anisotropic materials [21].
